# Lab tests can be used to predict phosphatidylethanol-measured high-risk alcohol use among people with HIV: a proof-of-concept using machine learning

**DOI:** 10.1093/alcalc/agaf078

**Published:** 2025-12-15

**Authors:** C Espinosa da Silva, A Scheffler, R Fatch, W Muyindike, N I Emenyonu, J Adong, G Chamie, C Ngabirano, A Kekibiina, A Tumwegamire, K Marson, B Beesiga, E Kindoli, I E Allen, J A Hahn, Drinkers’ Intervention to Prevent Tuberculosis (DIPT) Study and the Alcohol Drinkers’ Exposure to Preventive Therapy for TB (ADEPT-T) Study

**Affiliations:** Department of Medicine, University of California San Francisco, 550 16th Street, San Francisco, CA, 94158, USA; Department of Epidemiology and Biostatistics, University of California San Francisco; 550 16th Street, San Francisco, CA, 94158, USA; Department of Medicine, University of California San Francisco, 550 16th Street, San Francisco, CA, 94158, USA; Department of Medicine, Mbarara University of Science and Technology, Mbarara-Kabale Highway, Plot 8-18, Mbarara City, Uganda; Mbarara Regional Referral Hospital, Mbarara, Uganda; Department of Medicine, University of California San Francisco, 550 16th Street, San Francisco, CA, 94158, USA; Department of Medicine, Mbarara University of Science and Technology, Mbarara-Kabale Highway, Plot 8-18, Mbarara City, Uganda; Department of Medicine, University of California San Francisco, 550 16th Street, San Francisco, CA, 94158, USA; MUST Grants Office, Mbarara University of Science & Technology, Plot 8-18 Kabale Road, Mbarara, Uganda; MUST Grants Office, Mbarara University of Science & Technology, Plot 8-18 Kabale Road, Mbarara, Uganda; MUST Grants Office, Mbarara University of Science & Technology, Plot 8-18 Kabale Road, Mbarara, Uganda; Department of Medicine, University of California San Francisco, 550 16th Street, San Francisco, CA, 94158, USA; Infectious Diseases Research Collaboration, IDRC – Mbarara Office, Ntare Road, off Nkokonjeru Junction, Mbarara, Uganda; Infectious Diseases Research Collaboration, IDRC – Mbarara Office, Ntare Road, off Nkokonjeru Junction, Mbarara, Uganda; Department of Epidemiology and Biostatistics, University of California San Francisco; 550 16th Street, San Francisco, CA, 94158, USA; Department of Medicine, University of California San Francisco, 550 16th Street, San Francisco, CA, 94158, USA; Department of Epidemiology and Biostatistics, University of California San Francisco; 550 16th Street, San Francisco, CA, 94158, USA

**Keywords:** screening, detection, alcohol biomarkers, prediction tool

## Abstract

**Background:**

Unhealthy alcohol use is prevalent among persons with HIV (PWH) and is associated with adverse outcomes, but is underestimated partly due to use of self-reported measures prone to underreporting. Phosphatidylethanol (PEth) is a direct measure of past month alcohol consumption but is costly. We assessed whether lab and health data representing alcohol-associated physiologic changes could be leveraged with machine learning to predict PEth-measured high-risk alcohol use among PWH.

**Methods:**

We pooled baseline data from two studies among PWH in Uganda that measured PEth (N = 988), and classified PEth as no/low/moderate (PEth <200 ng/ml) or high-risk (PEth ≥200 ng/ml). We split the data into training (n = 790) and testing (n = 198) sets, imputing missing data separately for each. We conducted supervised learning with 29 predictors from lab (e.g. complete blood count, liver enzymes) and other health (e.g. age, sex, blood pressure) data using LASSO logistic regression, extreme gradient boosted decision trees, and random forests. We identified the optimal model via the largest area under the curve (AUC).

**Results:**

A LASSO regression with 17 predictors was the optimal model (cross-validated AUC in the Training Set = 0.751, 95% confidence interval [CI]: 0.718–0.784; AUC in Testing Set = 0.795, 95% CI: 0.723–0.852).

**Conclusions:**

This study suggests that a combination of lab and health data is useful for identifying individuals engaging in high-risk alcohol use (PEth ≥200 ng/ml). Algorithms including other indirect markers of alcohol use (e.g. gamma glutamyltransferase) may improve identification of high-risk alcohol use, which could be useful in research and clinical settings where PEth testing is unavailable.

## Introduction

Alcohol use is common globally and represents the 7^th^ leading risk factor for early death and disability (Griswold et al. 2018). Unhealthy alcohol use (i.e. consumption that could lead or has led to harmful psychological or physical outcomes [Donroe and Edelman 2022]) is prevalent among persons with HIV (PWH) and is associated with serious adverse outcomes including engaging in high-risk sexual behaviors, poor antiretroviral therapy (ART) adherence, increased HIV disease progression, various co-morbidities (e.g. cancer, liver cirrhosis, cardiovascular disease, neurocognitive impairment), increased susceptibility to infectious diseases, and premature mortality (Degenhardt et al. 2018, Probst et al. 2018, Duko et al. 2019, World Health Organization 2019, Saitz et al. 2021, Donroe and Edelman 2022, Lyu et al. 2022).

Increasing levels of alcohol use have been associated with physiological changes including increased levels of liver-derived enzymes, hematological alterations, and decreased levels of inflammatory markers (Niemelä 2016, Harris et al. 2021). For example, the liver is susceptible to alcohol-related damage given its influential role in metabolizing alcohol (Cederbaum 2012, Hyun et al. 2021, Thomes et al. 2021), with studies finding positive associations between the levels of alcohol use and alanine aminotransaminase (ALT), aspartate aminotransferase (AST), AST to ALT Ratio, and AST to Platelet Ratio Index (APRI) levels (Ferguson et al. 2020, Raabe et al. 2021, Høiseth et al. 2022). Moreover, numerous studies have found that alcohol consumption is positively associated with red blood cell volume (i.e. mean corpuscular volume) and mean corpuscular hemoglobin while negatively associated with red blood cell count, white blood cell count, lymphocyte count, leukocyte count, erythrocyte count, platelet count, hemoglobin, and hematocrit (Das et al. 2011, Thoma et al. 2015, Quraishi et al. 2016, Elanchezhian et al. 2017, Berad and Chand 2019, Wakabayashi 2021). These alcohol-associated physiological changes can potentially be leveraged to objectively identify PWH with high-risk alcohol consumption, defined by the World Health Organization as an average daily intake of >40 g/day for women and > 60 g/day for men (World Health Organization 2000).

Effective alcohol use screening among PWH could mitigate the morbidity and mortality associated with high-risk alcohol use. Self-reported measures are often used to assess alcohol use and are recommended for adult primary care visits (Curry et al. 2018), but are prone to underreporting due to many factors (e.g. recall issues, social desirability bias, alcohol-related stigma [Mooney et al. 2018, Kilian et al. 2021, Nielsen et al. 2021, Schell et al. 2021]). Direct measures of alcohol consumption exist, including the alcohol biomarker phosphatidylethanol (PEth) which is formed only after alcohol consumption and accumulates in red blood cells where it can be detected for several weeks (Luginbühl et al. 2022a). PEth values ≥200 ng/ml are considered indicative of high-risk alcohol use (Ulwelling and Smith 2018) or chronic excessive alcohol consumption (Luginbühl et al. 2022b). PEth is analyzed in specialized lab facilities using liquid chromatography with tandem mass spectrometry (LC–MS/MS) (Luginbühl et al. 2021), making it costly and infrequently implemented in current clinical practice or research settings. Given these limitations in current alcohol measurement strategies, high-risk alcohol use is often underestimated among PWH, resulting in a missed opportunity to engage at-risk individuals in strategies to reduce their alcohol use and improve their health outcomes.

Alcohol consumption is associated with unique physiological changes that individually may not accurately determine alcohol use, but could be leveraged in combination to create an objective screening tool for high-risk alcohol use. Certain lab tests (e.g. complete blood count) that are routinely collected in primary care settings may serve this purpose. Some authors have recommended using a combination of lab data to predict alcohol use, but did not specify how to combine measures (Andresen-Streichert et al. 2018, Ghosh et al. 2019).

In traditional statistics, a predictive model is developed to fit the data used to create the model implied which renders the model prone to overfitting and less robust to predicting future events in other samples (Bi et al. 2013). In comparison, machine learning offers a data-driven approach to combine measures that moves beyond traditional statistical estimation methods by constructing prediction models through an iterative process of learning from data patterns in one dataset, and subsequently validating and calibrating the optimal model using new data (Ebrahimi et al. 2021b). This approach allows for these models to better make clinical predictions (e.g. risk of alcohol use disorder or other poor health outcomes) and have greater generalizability in external samples (Ebrahimi et al. 2021b).

Using pooled data from two studies designed to include participants with high-risk alcohol use as well as those with little or no alcohol use, the goal of our exploratory study was to address a research gap by determining whether routine lab tests (e.g. complete blood count, liver enzymes) and other health data (e.g. age, sex, blood pressure) could be used to predict high-risk alcohol use detected by PEth ≥200 ng/ml (Ulwelling and Smith 2018, Luginbühl et al. 2022b) among PWH using machine learning approaches.

## Materials and methods

### Study population and design

We pooled available baseline data (N = 988) from two studies designed to include populations with a range of alcohol consumption, namely the Drinkers’ Intervention to Prevent Tuberculosis (DIPT) Study (Lodi et al. 2021) and the Alcohol Drinkers’ Exposure to Preventive Therapy for TB (ADEPT-T) study (Hahn et al. 2023). Baseline data were used because lab tests were not conducted at follow-up visits. For both studies, PEth testing was conducted at the United States Drug Testing Laboratory (USDTL, Des Plaines, IL, USA) and all other lab testing was conducted in the same lab in Uganda (facilitating comparability of the lab data between studies).

The DIPT Study was a randomized controlled trial (RCT) conducted in Uganda among individuals with HIV and latent TB who reported high-risk alcohol use (N = 687, NCT03492216) (Chamie et al. 2023). The aim of the RCT was to assess the efficacy of multiple incentive-based approaches to reduce alcohol use and enhance isoniazid medication adherence. Individuals were recruited in HIV clinics in one urban and two rural settings between 2018 and 2021. Individuals completed a screener using the Alcohol Use Disorders Identification Test – Consumption (AUDIT-C, past 3 months) and the alcohol biomarker urine ethyl glucuronide (EtG), and were eligible to participate in the study if they screened positive for current high-risk alcohol use (AUDIT-C ≥ 3 for women, AUDIT-C ≥4 for men, and EtG >300 ng/ml (Alcover et al. 2022)). Data from questionnaires and blood samples (via venous blood draw) were collected at baseline. Blood samples for PEth testing were spotted onto Whatman 903 paper, allowed to dry, then stored and shipped in batches to the United States Drug Testing Laboratory (USDTL, Des Plaines, IL, USA) with a limit of quantification of 8 ng/ml for the 16:0/18:1 analog (Jones et al. 2011). All other lab testing was conducted in a lab in Uganda. Additional study details have been published elsewhere (Lodi et al. 2021, Chamie et al. 2023).

The ADEPT-T study was conducted in Uganda among PWH (N = 301), and aimed to assess the incidence of isoniazid-related toxicity and whether this type of toxicity varied by the quantity of alcohol consumed. Individuals were recruited at local HIV clinics from 2017 to 2020. The study purposefully enrolled 101 participants without current alcohol use (≥1 year) and 200 participants reporting current alcohol use (<3 months). The blood samples for PEth testing were collected using the same procedures as the DIPT study, with PEth assays performed at USDTL. All other lab testing was conducted in the same lab in Uganda that was used for the DIPT Study. Additional details regarding study procedures are published elsewhere (Hahn et al. 2023).

In both studies, participants provided written informed consent. All study procedures were approved by the appropriate institutional review boards of ethics committees.

### Data collection

#### Outcome

We measured PEth from dried blood spots to determine alcohol use in the past month. As our study was a proof-of-concept, we classified participants using an established cut-off with no, low, or moderate alcohol use if PEth <200 ng/ml, and high-risk alcohol use if PEth values ≥200 ng/ml (Ulwelling and Smith 2018, Luginbühl et al. 2022b).

#### Predictors

In this secondary data analysis, we used available lab data which included: complete blood count with differential (i.e. red blood cell count, red cell distribution width, mean corpuscular volume, hemoglobin [g/dL], mean corpuscular hemoglobin, mean corpuscular hemoglobin concentration, percentage of hematocrit, white blood cell count [10^3^ μl], percentage of neutrophils, absolute count of neutrophils, percentage of eosinophils, absolute count of eosinophils, percentage of basophils, absolute count of basophils, percentage of lymphocytes, percentage of monocytes, platelet count [10^3^ μl], creatinine (mg/dL), AST, ALT, AST/ALT ratio, and other health data (i.e. age [years], biological sex [female, male], weight [kg], height [cm], body mass index [BMI, continuous and categorized as <18.5, 18.5 to <25, ≥25], systolic blood pressure [mmHg], diastolic blood pressure [mmHg], and Fibrosis-4 score).

### Statistical analysis

#### Sample characteristics

We generated descriptive statistics using medians for continuous variables and frequencies for categorical variables overall and stratified by study.

#### Predicting PEth-measured alcohol use using machine learning

We pooled the data from both studies into a single dataset (N = 988), and then randomly split the data into two independent sets: [1] a training set (n = 790, 80%) to develop the prediction models and perform cross-validation, and [2] a testing set for validation of the optimal model using new data (n = 198, 20%). We used Analysis of Variance (ANOVA) testing to confirm that the distribution of the predictors was comparable across the two data subsets.

To deal with missingness, we took the following steps. We assessed the degree of missingness, which ranged from less than 1% (e.g. BMI) to 36% (e.g. absolute count of eosinophils, Supplementary Table 1). We inspected missing data patterns, considered them to be likely missing at random, and imputed missing data separately for the training and test data with a single imputation via fully conditional specification using chained equations. While some authors demonstrated the utility of multiple imputation for predictive models (Sisk et al. 2023), the exact utility as well as how to pool results is not as well established. Given the proof-of-concept spirit of our model development, a single imputation was deemed sufficient. The imputation model developed in the training set was used to impute missing data in the testing set; the outcome of interest (i.e. PEth) was excluded from the imputation model. After data were imputed, we then standardized continuous variables to a mean of zero and a standard deviation of one. We used density plots to verify that the imputed values had a comparable distribution to the observed data.

Given that our study was a proof-of-concept, we wanted to compare the prediction models generated from an additive linear model (i.e. Least Absolute Shrinkage and Selection Operator [LASSO] logistic regression) versus two highly sophisticated non-linear machine learning approaches (i.e. extreme gradient boosted decision trees and random forests). The machine learning approaches considered are briefly described as follows. LASSO is a form of regularized regression that simultaneously predicts the outcome while performing variable selection by estimating associations for important predictors while shrinking the coefficients of noninfluential predictors to zero (James et al. 2021). A decision tree is a non-linear learning algorithm that classifies subjects into categories, using top-down, recursive partitioning (Subasi et al. 2022). Extreme gradient boosting is an ensemble learning approach that iteratively improves or ‘boosts’ the performance of a weak model by combining it with other weak models to develop an overall strong model with better predictive performance. Random forest is another non-linear learning algorithm that generates full decision trees in parallel and then averages the results of all the decision tree predictions (Subasi et al. 2022).

For each of these machine learning approaches (i.e. LASSO logistic regression, extreme gradient boosted decision trees, random forests), we implemented a two-stage model selection process (Kuhn and Johnson 2013) where we: (i) identified the best performing model in the training set (n = 790) based on which machine learning model had the largest cross-validated area under the curve (AUC) and the simplest structure, and (ii) evaluated the performance of this optimal model using new data in the testing set (n = 198). We considered AUC values instead of other model descriptors because it is a threshold-free metric that summarizes the performance of a classification model across all possible thresholds and, as such, provides a broad assessment of the classification model’s performance. When the optimal model from different machine learning algorithms (i.e. LASSO regression, extreme gradient boosted decision trees, random forests) yielded similar AUC values with overlapping confidence intervals, we preferred the algorithm with the simplest structure (i.e. LASSO regression versus more complex ensemble methods).

We used the *tidymodels* package (Version 1.1.0, Kuhn and Silge 2022) to develop our machine learning classification models and conducted k-fold cross-validation using the *vfold_cv* function, specifying 10 folds and one repeat of the k-fold partitioning. For the LASSO regression, we used the *logistic_reg* function with ‘glm’ as the estimation method. We then created a grid of tuning parameters using the *grid_regular* function (specifying 100 levels). For the extreme gradient boosted trees, we used the *boost_tree* function with ‘xgboost’ as the estimation method, and specifying 1000 trees. We used the *grid_latin_hypercube* function to develop a grid of tuning parameters and allowed for a maximum of 30 parameter value combinations. For the random forests, we used the *rand_forest* function with ‘ranger’ as the estimation method. We used the *grid_regular* function to generate a grid of tuning parameters, indicating a range of 100–1000 trees and 2–20 predictors to be randomly sampled at each split when building the decision tree. Across all machine learning approaches (i.e. LASSO logistic regression, extreme gradient boosted decision trees, random forests), we then conducted model tuning by evaluating lambda values. To identify the optimal lambda value, we used the *collect_metrics* function to plot cross-validated performance metrics and selected the model with the highest AUC value. We then used the *ci_auc* function in the *pROC* package (Version 1.18.5, Robin et al. 2021) to bootstrap confidence intervals for the AUC value of the optimal model.

As we considered it important to rank individuals by risk, we generated a receiver operating characteristic (ROC) curve to assess the discriminatory performance of each cross-validated optimal model from our three selected machine learning approaches (i.e. LASSO logistic regression, extreme gradient boosted decision trees, random forests). These ROC curves plot the true positive rate (i.e. sensitivity) by the false positive rate (i.e. 1 – specificity), with a larger area under the ROC curve indicating better model prediction performance. We also assessed model calibration using calibration plots (using the *cal_plot_breaks* function in the *tidymodels* package, Kuhn and Silge 2022) and the Integrated Calibration Index (ICI) to understand the accuracy of the predicted probabilities of high-risk alcohol use. We then assessed the predictive performance of the optimal model in the testing data (n = 198).

As a post-hoc analysis, we estimated the predictive performance of a logistic regression modeling high-risk alcohol use (PEth ≥200 ng/ml) as a function of self-reported alcohol use (AUDIT-C continuous score 0–12) for comparison with the results from our optimal machine learning model.

We report our machine learning model development and validation using the TRIPOD guidelines (Collins et al. 2015). All analyses were conducted using STATA (Version 17.0) or R (Version 4.3.1).

## Results

In our overall sample (N = 988), participants were a median age of 40 years (interquartile range [IQR] = 32–47), 63% were male, and the prevalence of PEth ≥200 ng/ml was 46% (Table 1). Participants in the DIPT RCT had higher PEth levels compared to those in the ADEPT-T study (median 248 ng/ml versus 17 ng/ml, respectively), which was expected given their different study eligibility criteria.

**Table 1:** Participant characteristics across predictor variables used in machine learning models in the training set, median [IQR]

Characteristics		Overall Sample (N = 790)	DIPT (n = 545)	ADEPT-T (n = 245)
PEth (ng/ml)		167 [23, 467]	248 [80, 549]	17 [1, 194]
No, low, or moderate alcohol use (<200 ng/ml), n(%)		430 (54)	246 (45)	184 (75)
High-risk use (≥200 ng/ml), n(%)		360 (46)	299 (55)	61 (25)
**Complete Blood Count with Differential Panel**				
1	Red blood cell count	4.6 [4.2, 5.1]	4.6 [4.2, 5.1]	4.6 [4.2, 5.0]
2	Red cell distribution width	12.2 [10.8, 13.3]	11.1 [9.9, 12.6]	12.8 [12.0, 13.6]
3	Mean corpuscular volume	94.9 [89.0, 100.0]	96.0 [90.0, 100.3]	91.2 [86.0, 97.9]
4	Hemoglobin (g/dL)	14.5 [13.3, 15.7]	14.6 [13.3, 15.9]	14.5 [13.4, 15.6]
5	Mean corpuscular hemoglobin	31.6 [29.6, 33.4]	31.7 [29.8, 33.4]	31.5 [29.2, 33.3]
6	Mean corpuscular hemoglobin concentration	33.3 [32.0, 34.8]	33.1 [31.7, 34.6]	33.6 [32.5, 35.4]
7	Percentage of hematocrit	43.4 [40.0, 47.1]	43.9 [40.6, 47.7]	42.8 [39.3, 45.4]
8	White blood cell count (10^3^ μl)	4.6 [3.8, 5.5]	4.7 [3.8, 5.7]	4.4 [3.8, 5.1]
9	Percentage of neutrophils	52.4 [44.2, 59.9]	54.0 [45.6, 60.3]	49.3 [42.2, 56.8]
10	Absolute count of neutrophils	2.3 [1.8, 3.1]	2.4 [1.9, 3.3]	2.2 [1.7, 2.7]
11	Percentage of eosinophils	2.0 [1.1, 3.8]	1.8 [1.0, 3.6]	2.1 [1.2, 3.9]
12	Absolute count of eosinophils	0.1 [0.1, 0.2]	0.1 [0.1, 0.2]	0.1 [0.1, 0.2]
13	Percentage of basophils	0.7 [0.4, 1.1]	0.8 [0.6, 1.4]	0.5 [0.3, 0.9]
14	Absolute count of basophils	0.0 [0.0, 0.1]	0.0 [0.0, 0.1]	0.0 [0.0, 0.0]
15	Percentage of lymphocytes	36.1 [29.7, 43.0]	35.1 [28.6, 42.0]	38.4 [32.2, 45.1]
16	Percentage of monocytes	7.7 [5.8, 9.9]	8.0 [5.8, 10.2]	7.4 [5.6, 9.0]
17	Platelet count (10^3^ μl)	237.0 [194.0, 288.0]	233.0 [189.0, 284.0]	250.0 [206.0, 297.0]
**Comprehensive Metabolic Panel**				
18	Creatinine (mg/dL)	0.8 [0.7, 1.0]	0.8 [0.6, 1.0]	0.8 [0.7, 1.0]
19	Aspartate aminotransferase (AST)	33.3 [27.0, 43.7]	34.0 [27.3, 45.2]	32.0 [25.6, 39.1]
20	Alanine aminotransaminase (ALT)	27.5 [20.7, 38.0]	27.1 [20.3, 38.0]	28.4 [22.0, 38.0]
21	AST/ALT Ratio	1.2 [1.0, 1.5]	1.3 [1.0, 1.6]	1.1 [0.9, 1.4]
**Other Health Data**				
22	Age (years)	40 [32, 47]	40 [32, 47]	40 [34, 47]
23	Biological sex, n(%)			
Female		292 (37)	169 (31)	123 (50)
Male		498 (63)	376 (69)	122 (50)
24	Weight (kg)	59.0 [53.7, 67.0]	58.0 [52.0, 65.0]	63.0 [56.4, 71.2]
25	Height (cm)	165.6 [159.4, 171.5]	166.0 [159.6, 171.5]	165.5 [159.2, 172.2]
26	Body mass index	21.6 [19.2, 24.1]	20.8 [18.8, 23.4]	22.6 [20.3, 25.4]
< 18.5 kg/m^2^, n(%)		142 (18)	116 (21)	26 (11)
18.5 to <25 kg/m^2^, n(%)		488 (62)	339 (62)	149 (61)
≥ 25 kg/m^2^, n(%)		159 (20)	89 (16)	70 (29)
27	Systolic blood pressure (mmHg)	117 [108, 125]	118 [109, 125]	117 [107, 126]
28	Diastolic blood pressure (mmHg)	75 [69, 81]	76 [69, 83]	73 [67, 80]
29	Fibrosis-4	1.1 [0.7, 1.5]	1.2 [0.8, 1.7]	1.0 [0.7, 1.4]

Abbreviations: ADEPT-T = the Alcohol Drinkers’ Exposure to Preventive Therapy for TB (ADEPT-T) study, DIPT = the Drinkers’ Intervention to Prevent Tuberculosis RCT

**Figure 1:**
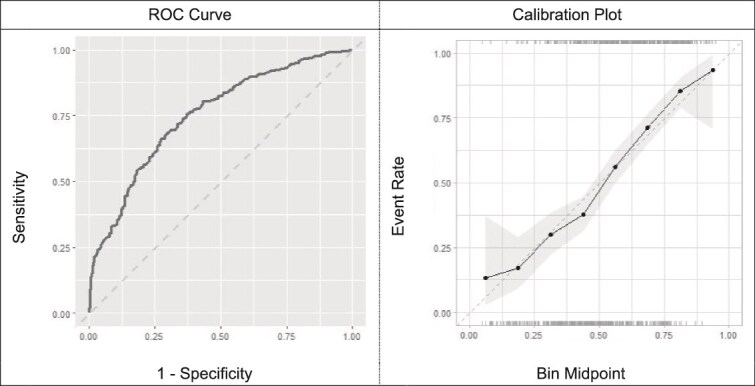
Cross-validated discrimination and calibration of optimal model developed using Least Absolute Shrinkage and Selection Operator (LASSO) logistic regression and selected predictors in the training set (n = 790)

**Figure 2:**
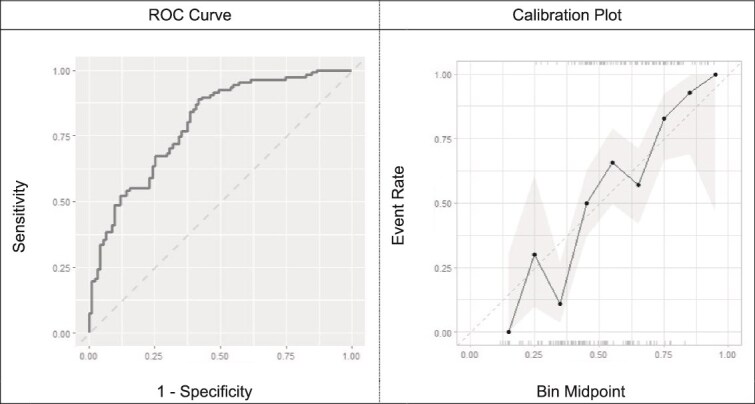
Cross-validated discrimination and calibration of optimal model developed using Least Absolute Shrinkage and Selection Operator (LASSO) logistic regression and selected predictors using new data in the testing set (n = 198)

In the training set (n = 790), optimal models developed using LASSO logistic regression, extreme boosted decision trees, and random forests had comparable cross-validated AUC values of 0.751 (95% CI = 0.718–0.784), 0.748 (95% CI = 0.710–0.781), and 0.734 (95% CI = 0.699–0.769), respectively (Table 2). Given the overlapping confidence intervals and the lack of evidence of superior fit for the more complex models (i.e. extreme gradient boosted decision trees and random forests), we used Occam’s razor (Blumer et al. 1987) and considered the LASSO logistic regression model with 17 variables to be the optimal model since it had the highest AUC value and the simplest structure (ICI: 0.020, Fig. 1). Based on the optimal hyperparameter settings identified via cross-validation, a final LASSO model was estimated on all the training data. In the testing set (n = 198), which was independent of the training set and contained new data not used to develop the prediction models), our optimal model displayed comparable discriminatory performance compared to the cross-validated results, achieving an AUC value of 0.795 (95% CI = 0.723–0.852, ICI, 0.065, Fig. 2). Within this model in the testing set, variables positively associated with high-risk alcohol use (PEth ≥200 ng/ml) included AST, diastolic blood pressure, mean corpuscular hemoglobin, platelet count, percentage of hematocrit, percentage of monocytes, and mean corpuscular volume; predictors negatively associated with PEth ≥200 ng/ml included female sex, BMI (continuous), ALT, red cell distribution width, weight, white blood cell count, percentage of lymphocytes, age, absolute count of eosinophils, and BMI ≥ 25 kg/m^2^.

**Table 2:** Predictive performance of different machine learning algorithms to estimate high-risk alcohol use among people with HIV using a two-step model selection approach

Machine Learning Algorithm	Training Set (n = 790)		Testing Set (n = 198)	
	AUC Value	95% CI	AUC Value	95% CI
LASSO Logistic Regression	0.751	0.718–0.784	0.795	0.723–0.852
Extreme Gradient Boosted Decision Trees	0.748	0.710–0.781	-	-
Random Forests	0.734	0.699–0.769	-	-

Abbreviations: AUC = area under the curve, CI = confidence interval, LASSO = Least Absolute Shrinkage and Selection Operator

In our post-hoc analysis, a logistic regression modeling high-risk alcohol use as a function of self-reported alcohol use (AUDIT-C continuous score) yielded AUC values of 0.724 (95% CI = 0.689–0.758) and 0.698 (95% CI = 0.626–0.769) in the training and testing sets, respectively.

## Discussion

We found high proof-of-concept of using lab tests and health data to build machine learning prediction models for PEth-measured high-risk alcohol use (PEth ≥200 ng/ml) among PWH (Ulwelling and Smith 2018, Luginbühl et al. 2022b). We found that in 80% of cases our optimal model would correctly identify an individual with high-risk alcohol use. The optimal models identified from three different machine learning approaches were comparable. This study builds upon previous work implementing machine learning algorithms to predict alcohol use (O'Halloran et al. 2018, Ngo et al. 2019, Bonnell et al. 2020, Ebrahimi et al. 2020, Gowin et al. 2021, Kim et al. 2021, Ebrahimi et al. 2021a, Pinar-Sanchez et al. 2022) by utilizing a direct measure of alcohol consumption (i.e. PEth) as the outcome, while most of the previous studies used self-reported or other indirect measures of alcohol use (e.g. self-reported AUDIT-C, harmful drinking events [O'Halloran et al. 2018, Ngo et al. 2019, Bonnell et al. 2020, Ebrahimi et al. 2020, Gowin et al. 2021, Kim et al. 2021, Ebrahimi et al. 2021a]).

Our study was proof-of-concept and opportunistically used available lab measures that were collected for other research purposes. Future work developing machine learning prediction models of alcohol use among PWH would benefit from assessing the value of other lab measures (e.g. gamma glutamyltransferase, lipids), which were not available for this study. Nevertheless, our findings have important implications towards the eventual development of a predictive tool to better detect alcohol use in this population. In clinical settings, this type of a prediction tool could serve as a first-line screening of alcohol use, which is also typically the most labor-intensive; the algorithms from these machine learning models could be integrated in medical health systems to generate predictions of alcohol use from routine lab data, and subsequently flag the medical records of PWH who may benefit from further clinical resources (Bonnell et al. 2020). The implications of our findings are also highly pertinent in research settings in which assessing alcohol use is important but may be susceptible to the biases inherent in self-report (Mooney et al. 2018, Kilian et al. 2021, Nielsen et al. 2021, Schell et al. 2021). Prediction models based on self-reported alcohol use may tend to be specific to certain populations and settings as they are susceptible to self-report biases that differ across cultures (e.g. social desirability in different settings associated with under-reporting of alcohol use [Adong et al. 2019]); on the other hand, prediction models developed using biological predictor data are not subject to self-report bias and may offer a better prediction tool for high-risk alcohol use across populations. While many studies may be unable to collect costly alcohol biomarker data and historical repositories lack samples needed for PEth testing (whole blood or DBS), our findings suggest that more affordable and existing lab and health data may be used in conjunction with machine learning methods to create an objective measure of high-risk alcohol consumption. This algorithm could identify which lab data are useful and which data are unnecessary given their minimal impact on predicting high-risk alcohol use, and leveraging machine learning would improve generalizability compared to prediction models generated from traditional statical methods (Ebrahimi et al. 2021b).

Our study has some limitations. Our study sample was comprised of PWH living in Uganda, and may not be representative of PWH in other settings. Future work should develop machine learning prediction models for alcohol use among PWH in other contexts (e.g. high-resource settings) and in larger, more generalizable samples. We implemented single imputation, which is prone to underestimating the variance associated with missing values compared to more sophisticated imputation methods. While this choice may have impacted the point prediction and confidence intervals of the AUC values for candidate models during model selection, we considered this acceptable given that the aim of our study was to quantify the predictive performance of our model in the testing set. In other words, using a single imputation potentially made our model selection process less than optimal by ranking a suboptimal model ahead of a superior model. However, the assessment of our final model on the testing set remains an unbiased estimate of the generalizability error of the whole procedure and is unaffected by the choice of single versus multiple imputation. This is because the imputation procedure, much like the fitting of the actual model, was informed exclusively by the training data. We dichotomized the PEth outcome variable which likely oversimplified the data and resulted in a loss of information regarding the spectrum of alcohol use. However, this categorization was necessary to develop classification models and our dichotomization threshold was informed by a recommended cut-off value (PEth ≥200 ng/ml) that is indicative of high-risk alcohol use. Our predictive models were restricted to the tests collected in two laboratory panels (e.g. complete blood count, comprehensive metabolic panel) and limited health data relevant to the parent study research. Future studies should build on our work and incorporate predictors from a wider range of lab tests known to be affected by alcohol consumption to assess whether the predictive value of the machine learning algorithms improves. Our predictive models did not consider confounding control because our work was not centered on causal inference nor disentangling the causal relationships between variables of interest; instead, our primary objective was to determine whether specific independent variables could accurately predict biomarker-measured high-risk alcohol use. Unmeasured confounding may reduce the generalizability of our prediction modeling if the confounding relationships in our sample change significantly in external samples. However, cross-validation is an approach that we implemented to examine the predictive ability of the optimal models in scenarios using different data. Our work is strengthened by modeling an alcohol biomarker (i.e. PEth) as our outcome measure and the use of other lab and health data predictors (compared to machine learning models using self-reported data as outcome and predictor variables), as well as the use of pooled data from two different studies whose study populations reported a range of alcohol consumption.

This proof-of-concept study was a first exploration of the discriminative and absolute performance of a machine learning prediction model for PEth-measured high-risk alcohol use. Our findings suggest that using lab tests and other health data may be useful in predicting high-risk alcohol use among PWH. Future studies should conduct these analyses in a larger sample with more lab tests and health data to assess any improvements in classification. Future work should also explore the ability of lab and health data to differentiate between light and moderate alcohol use. Additional research is also needed exploring cost benefit-analysis via decision curves (Zhang et al. 2018, Vickers et al. 2019).

## Supplementary Material

20251111_PilotML_SupplT1_agaf078

## Data Availability

The data used for these analyses are available on request from the senior author, Dr. Judy Hahn.

## References

[ref1] Adong J, Fatch R, Emenyonu NI. et al. Social desirability bias impacts self-reported alcohol use among persons with HIV in Uganda. Alcohol Clin Exp Res. 2019;43:2591–8. 10.1111/acer.14218 31610017 PMC7411366

[ref2] Alcover KC, Emenyonu NI, Fatch R. et al. Concordance between point-of-care urine ethyl glucuronide alcohol tests and self-reported alcohol use in persons with HIV in Uganda. AIDS Behav. 2022;26:2539–47. 10.1007/s10461-022-03597-6 35103888 PMC9256760

[ref3] Andresen-Streichert H, Müller A, Glahn A. et al. Alcohol biomarkers in clinical and forensic contexts. Dtsch Arztebl Int. 2018;115:309. 29807559 10.3238/arztebl.2018.0309 PMC5987059

[ref4] Berad A, Chand V. Study to compare hematological parameters in alcoholic and non-alcoholic individuals. National Journal of Physiology, Pharmacy and Pharmacology. 2019;9:1176–6.

[ref5] Bi J, Sun J, Wu Y. et al. A machine learning approach to college drinking prediction and risk factor identification. ACM Transactions on Intelligent Systems and Technology (TIST). 2013;4:1–24. 10.1145/2508037.2508053

[ref6] Blumer A, Ehrenfeucht A, Haussler D. et al. Occam's razor. Occam's razor Information processing letters. 1987;24:377–80. 10.1016/0020-0190(87)90114-1

[ref7] Bonnell LN, Littenberg B, Wshah SR. et al. A machine learning approach to identification of unhealthy drinking. The Journal of the American Board of Family Medicine. 2020;33:397–406. 10.3122/jabfm.2020.03.190421 32430371

[ref8] Cederbaum AI . Alcohol metabolism. Clin Liver Dis. 2012;16:667–85. 10.1016/j.cld.2012.08.002 23101976 PMC3484320

[ref9] Chamie G, Hahn JA, Kekibiina A. et al. Financial incentives for reduced alcohol use and increased isoniazid adherence during tuberculosis preventive therapy among people with HIV in Uganda: An open-label, factorial randomised controlled trial. In: *Lancet Global Health*, Vol. 11, 2023, e1899–910. 10.1016/S2214-109X(23)00436-9. 37973340 PMC11005200

[ref11] Collins GS, Reitsma JB, Altman DG. et al. Transparent reporting of a multivariable prediction model for individual prognosis or diagnosis (TRIPOD) the TRIPOD statement. Circulation. 2015;131:211–9. 10.1161/CIRCULATIONAHA.114.014508 25561516 PMC4297220

[ref12] Curry SJ, Krist AH, Owens DK. et al. Screening and behavioral counseling interventions to reduce unhealthy alcohol use in adolescents and adults: US preventive services task force recommendation statement. Jama. 2018;320:1899–909. 10.1001/jama.2018.16789 30422199

[ref13] Das S, Mukherjee S, Vasudevan D. et al. Comparison of haematological parameters in patients with non-alcoholic fatty liver disease and alcoholic liver. Singapore Med J. 2011;52:175–81. 21451926

[ref14] Degenhardt L, Charlson F, Ferrari A. et al. The global burden of disease attributable to alcohol and drug use in 195 countries and territories, 1990–2016: a systematic analysis for the global burden of disease study 2016. Lancet Psychiatry. 2018;5:987–1012. 10.1016/S2215-0366(18)30337-7 30392731 PMC6251968

[ref15] Donroe JH, Edelman EJ. Alcohol use. Ann Intern Med. 2022;175:ITC145-ITC160. 10.7326/AITC202210180 36215713

[ref16] Duko B, Ayalew M, Ayano G. The prevalence of alcohol use disorders among people living with HIV/AIDS: a systematic review and meta-analysis. Subst Abuse Treat Prev Policy. 2019;14:1–9. 31727086 10.1186/s13011-019-0240-3 PMC6854786

[ref17] Ebrahimi A, Wiil UK, Andersen K. et al. A predictive machine learning model to determine alcohol use disorder. In: 2020 IEEE Symposium on Computers and Communications (ISCC). IEEE, 2020, 1–7.

[ref18] Ebrahimi A, Wiil UK, Mansourvar M. et al. Deep neural network to identify patients with alcohol use disorder. MIE. 2021a;238–42. 10.3233/SHTI210156 34042741

[ref19] Ebrahimi A, Wiil UK, Schmidt T. et al. Predicting the risk of alcohol use disorder using machine learning: a systematic literature review. IEEE Access. 2021b;9:151697–712. 10.1109/ACCESS.2021.3126777

[ref20] Elanchezhian YT, Mayilsamy S, Radhakrishnan S. Comparison of haematological parameters between alcoholics and non-alcoholics. Int J Res Med Sci. 2017;5:5041–7.

[ref21] Ferguson TF, Rosen E, Carr R. et al. Associations of liver disease with alcohol use among people living with HIV and the role of hepatitis C: the New Orleans alcohol use in HIV study. Alcohol Alcohol. 2020;55:28–36. 10.1093/alcalc/agz089 31812989 PMC7005833

[ref22] Ghosh S, Jain R, Jhanjee S. et al. Alcohol biomarkers and their relevance in detection of alcohol consumption in clinical settings. Int Arch Subst Abuse Rehabil. 2019;1.

[ref23] Gowin JL, Manza P, Ramchandani VA. et al. Neuropsychosocial markers of binge drinking in young adults. Mol Psychiatry. 2021;26:4931–43. 10.1038/s41380-020-0771-z 32398720 PMC7658012

[ref24] Griswold MG, Fullman N, Hawley C. et al. Alcohol use and burden for 195 countries and territories, 1990–2016: a systematic analysis for the global burden of disease study 2016. The Lancet. 2018;392:1015–35. 10.1016/S0140-6736(18)31310-2 PMC6148333 30146330

[ref25] Hahn JA, Ngabirano C, Fatch R. et al. Safety and tolerability of isoniazid preventive therapy for tuberculosis for persons with HIV with and without alcohol use: a single arm trial. AIDS (London, England). 2023;37:1535–43. 10.1097/QAD.0000000000003613 37260251 PMC10355800

[ref26] Harris JC, Leggio L, Farokhnia M. Blood biomarkers of alcohol use: a scoping review. Curr Addict Rep. 2021;1–9. 10.1007/s40429-021-00402-7 PMC10237590 37274945

[ref27] Høiseth G, Hilberg T, Trydal T. et al. The alcohol marker phosphatidylethanol is closely related to AST, GGT, ferritin and HDL-C. Basic Clin Pharmacol Toxicol. 2022;130:182–90. 10.1111/bcpt.13662 34591374

[ref28] Hyun J, Han J, Lee C. et al. Pathophysiological aspects of alcohol metabolism in the liver. Int J Mol Sci. 2021;22:5717. 10.3390/ijms22115717 34071962 PMC8197869

[ref29] James G, Witten D, Hastie T. et al. *An introduction to statistical learning: With applications in R*. In: *An Introduction to Statistical Learning: With Applications in R*. Springer, 2021. 10.1007/978-1-0716-1418-1.

[ref30] Jones J, Jones M, Plate C. et al. The detection of 1-palmitoyl-2-oleoyl-sn-glycero-3-phosphoethanol in human dried blood spots. Anal Methods. 2011;3:1101–6. 10.1039/c0ay00636j

[ref31] Kilian C, Manthey J, Carr S. et al. Stigmatization of people with alcohol use disorders: an updated systematic review of population studies. Alcohol Clin Exp Res. 2021;45:899–911. 10.1111/acer.14598 33970504

[ref32] Kim S-Y, Park T, Kim K. et al. A deep learning algorithm to predict hazardous drinkers and the severity of alcohol-related problems using K-NHANES. Front Psych. 2021;12:684406. 10.3389/fpsyt.2021.684406 PMC8299053 34305681

[ref33] Kuhn M, Johnson K. *Applied Predictive Modeling*. Springer, 2013. 10.1007/978-1-4614-6849-3.

[ref34] Kuhn M, Silge J. *Tidy Modeling with R*. O'Reilly Media, Inc, 2022.

[ref35] Lodi S, Emenyonu NI, Marson K. et al. The drinkers’ intervention to prevent tuberculosis (DIPT) trial among heavy drinkers living with HIV in Uganda: study protocol of a 2 × 2 factorial trial. Trials. 2021;22:355. 10.1186/s13063-021-05304-7 34016158 PMC8136096

[ref36] Luginbühl M, Stöth F, Schröck A. et al. Quantitative determination of phosphatidylethanol in dried blood spots for monitoring alcohol abstinence. Nat Protoc. 2021;16:283–308. 10.1038/s41596-020-00416-x 33288956

[ref37] Luginbühl M, Van Uytfanghe K, Stöth F. et al. Current evolutions, applications, and challenges of phosphatidylethanol analysis for clinical and forensic purposes. Wiley Interdisciplinary Reviews: Forensic Science. 2022a;4:e1456.

[ref38] Luginbühl M, Wurst FM, Stöth F. et al. Consensus for the use of the alcohol biomarker phosphatidylethanol (PEth) for the assessment of abstinence and alcohol consumption in clinical and forensic practice (2022 consensus of Basel). Drug Test Anal. 2022b;14:1800–2. 10.1002/dta.3340 35851997

[ref39] Lyu H, Tang H, Liang Y. et al. Alcohol consumption and risk of liver fibrosis in people living with HIV: a systematic review and meta-analysis. Front Immunol. 2022;13:841314. 10.3389/fimmu.2022.841314 35371091 PMC8971654

[ref40] Mooney AC, Campbell CK, Ratlhagana M-J. et al. Beyond social desirability bias: investigating inconsistencies in self-reported HIV testing and treatment behaviors among HIV-positive adults in north West Province, South Africa. AIDS Behav. 2018;22:2368–79. 10.1007/s10461-018-2155-9 29779162 PMC13310556

[ref41] Ngo DA, Rege SV, Ait-Daoud N. et al. Development and validation of a risk predictive model for student harmful drinking—a longitudinal data linkage study. Drug Alcohol Depend. 2019;197:102–7. 10.1016/j.drugalcdep.2019.01.016 30802733

[ref42] Nielsen DG, Andersen K, Nielsen AS. et al. Consistency between self-reported alcohol consumption and biological markers among patients with alcohol use disorder–a systematic review. Neuroscience & Biobehavioral Reviews. 2021;124:370–85. 10.1016/j.neubiorev.2021.02.006 33581224

[ref43] Niemelä O . Biomarker-based approaches for assessing alcohol use disorders. Int J Environ Res Public Health. 2016;13:166. 10.3390/ijerph13020166 26828506 PMC4772186

[ref44] O'Halloran L, Pennie B, Jollans L. et al. A combination of impulsivity subdomains predict alcohol intoxication frequency. Alcohol Clin Exp Res. 2018;42:1530–40. 10.1111/acer.13779 29905967

[ref45] Pinar-Sanchez J, Bermejo López P, Solís García Del Pozo J. et al. Common laboratory parameters are useful for screening for alcohol use disorder: designing a predictive model using machine learning. J Clin Med. 2022;11:2061. 10.3390/jcm11072061 35407669 PMC8999878

[ref46] Probst C, Parry CD, Rehm J. HIV/AIDS mortality attributable to alcohol use in South Africa: a comparative risk assessment by socioeconomic status. BMJ Open. 2018;8:e017955. 10.1136/bmjopen-2017-017955 PMC5855363 29467131

[ref47] Quraishi R, Jain R, Ambekar A. Hematological profile of alcohol dependent subjects: report from a tertiary car e tceatment Centre in India. Int J Pharma Res Health Sci. 2016;4:1420–3. 10.21276/ijprhs.2016.05.15

[ref48] Raabe FJ, Wagner E, Weiser J. et al. Classical blood biomarkers identify patients with higher risk for relapse 6 months after alcohol withdrawal treatment. Eur Arch Psychiatry Clin Neurosci. 2021;271:891–902. 10.1007/s00406-020-01153-8 32627047 PMC8236027

[ref49] Robin X, Turck N, Hainard A. et al. Package pROC. Package pROC. 2021.

[ref50] Saitz R, Miller SC, Fiellin DA. et al. Recommended use of terminology in addiction medicine. J Addict Med. 2021;15:3–7. 10.1097/ADM.0000000000000673 32482955

[ref51] Schell C, Godinho A, Cunningham JA. To thine own self, be true: examining change in self-reported alcohol measures over time as related to socially desirable responding bias among people with unhealthy alcohol use. Subst Abus. 2021;42:87–93. 10.1080/08897077.2019.1697998 32040383

[ref52] Sisk R, Sperrin M, Peek N. et al. Imputation and missing indicators for handling missing data in the development and deployment of clinical prediction models: a simulation study. Stat Methods Med Res. 2023;32:1461–77. 10.1177/09622802231165001 37105540 PMC10515473

[ref53] Subasi A, Panigrahi SS, Patil BS. et al. Advanced pattern recognition tools for disease diagnosis. In: *5G IoT and Edge Computing for Smart Healthcare*. Elsevier, 2022. 10.1016/B978-0-323-90548-0.00011-5.

[ref54] Thoma E, Bitri S, Mucaj K. et al. Changes of some blood count variables in correlation with the time of alcohol abuse. J Addict Res Ther. 2015;6:2.

[ref55] Thomes PG, Rasineni K, Saraswathi V. et al. Natural recovery by the liver and other organs after chronic alcohol use. Alcohol Res. 2021;41. 10.35946/arcr.v41.1.05 PMC8041137 33868869

[ref56] Ulwelling W, Smith K. The PEth blood test in the security environment: what it is; why it is important; and interpretative guidelines. J Forensic Sci. 2018;63:1634–40. 10.1111/1556-4029.13874 30005144

[ref57] Vickers AJ, Van Calster B, Steyerberg EW. A simple, step-by-step guide to interpreting decision curve analysis. Diagnostic and prognostic research. 2019;3:1–8. 31592444 10.1186/s41512-019-0064-7 PMC6777022

[ref58] Wakabayashi I . Habitual alcohol drinking and peripheral blood cell counts in men with diabetes. Clinical Diabetology. 2021;10:484–8.

[ref59] World Health Organization . International Guide for Monitoring Alcohol Consumption and Related Harm, 2000.

[ref60] World Health Organization . *Global Status Report on Alcohol and Health 2018*. World Health Organization, 2019.

[ref61] Zhang Z, Rousson V, Lee W-C. et al. Decision curve analysis: a technical note. Annals of translational medicine. 2018;6:308. 10.21037/atm.2018.07.02 30211196 PMC6123195

