# Supplementary material for: Lab tests can be used to predict phosphatidylethanol-measured high-risk alcohol use among people with HIV: a proof-of-concept using machine learning
Source: Alcohol Alcohol. 2025 Dec 15;61(1):agaf078. doi: 10.1093/alcalc/agaf078 (PMC12704430; doi:10.1093/alcalc/agaf078)
Supplement: 20251111_PilotML_SupplT1_agaf078 [file 20251111_pilotml_supplt1_agaf078.pdf]

Supplementary Table 1. Percentage of missingness across predictor variables in the training set (N=790)

Characteristics	Missing Observations (%)
**Complete Blood Count with Differential Panel**	
Red blood cell count	0 (0)
Red cell distribution width	277 (35)
Mean corpuscular volume	0 (0)
Hemoglobin (g/dL)	0 (0)
Mean corpuscular hemoglobin	0 (0)
Mean corpuscular hemoglobin concentration	0 (0)
Percentage of hematocrit	0 (0)
White blood cell count (10^3 µL)	0 (0)
Percentage of neutrophils	7 (1)
Absolute count of neutrophils	7 (1)
Percentage of eosinophils	284 (36)
Absolute count of eosinophils	284 (36)
Percentage of basophils	278 (35)
Absolute count of basophils	278 (35)
Percentage of lymphocytes	0 (0)
Percentage of monocytes	0 (0)
Platelet count (10^3 µL)	0 (0)
**Comprehensive Metabolic Panel**	
Creatinine (mg/dL)	2 (<1)
Aspartate aminotransferase (AST)	0 (0)
Alanine aminotransaminase (ALT)	0 (0)
AST/ALT Ratio	0 (0)
**Other Health Data**	
Age (years)	0 (0)
Biological sex, n(%)	0 (0)
Weight (kg)	0 (0)
Height (cm)	0 (0)
Body mass index	1 (<1)
Systolic blood pressure (mmHg)	0 (0)
Diastolic blood pressure (mmHg)	0 (0)
Fibrosis-4	0 (0)
